# Multifactorial Analysis Identifies Conditions for Robust HCoV-OC43 Replication in Primary Human Bronchial Epithelial Cells Cultured at Air–Liquid Interface

**DOI:** 10.3390/cells15111010

**Published:** 2026-05-30

**Authors:** Natalie Fischhaber, Julian Vogler, Ivana Martan, Thomas Michler

**Affiliations:** Institute of Laboratory Medicine, Ludwig-Maximilians-University Hospital, Ludwig-Maximilians-University Munich, 80539 Munich, Germany; julian.vogler@med.uni-muenchen.de (J.V.);

**Keywords:** bronchial epithelial differentiation, Design of Experiment, HCOV-OC43, air–liquid interface

## Abstract

Air–liquid interface (ALI) cultures recapitulate key features of the airway epithelium by driving basal cell differentiation into ciliated, club, and goblet cells and by generating a functional mucus barrier, thereby representing a highly relevant model of the human respiratory tract. Using a reduced factorial Design of Experiments (DoE) methodology, we simultaneously investigated the effects of seven variables on human coronavirus OC43 (HCoV-OC43) replication in air–liquid interface (ALI)-cultured primary human bronchial epithelial cells (pHBECs) to identify robust conditions that support infection and viral replication. Epithelial differentiation was monitored by measuring transepithelial electrical resistance and determining expression levels of marker genes for basal, goblet, club, and ciliated cells using reverse transcription quantitative PCR (RT-qPCR). HCoV-OC43 replication was monitored by quantifying genomic and subgenomic RNA by RT-qPCR. Viral RNA peaked three days post-infection in cell lysates and four days post-infection in apical washes. Initiation of ALI conditions induced epithelial differentiation, which was complete after 21 days and emerged as the strongest determinant of viral replication. Differentiated pHBEC cultures showed significantly reduced viral RNA compared with undifferentiated cultures, particularly following apical infection. In contrast, basal infection resulted in lower viral RNA levels in undifferentiated cultures than apical infection but was less dependent on epithelial differentiation. However, productive infection following basal exposure was less consistent and more strongly dependent on viral inoculum size. We further demonstrate that repeated mucus washes prior to infection increased HCoV-OC43 replication in mature cultures. In summary, our findings show that epithelial differentiation negatively affects HCoV-OC43 replication and we identify conditions that maximize viral replication in fully differentiated pHBEC cultures.

## 1. Introduction

Coronaviruses (CoVs) are a large family of enveloped, positive-sense, single-stranded RNA viruses that infect a wide range of vertebrates, including mammals and birds [1,2]. Multiple zoonotic spillovers in the last decades have led to the emergence of the highly pathogenic betacoronaviruses severe acute respiratory syndrome (SARS)-CoV, SARS-CoV-2 and middle east respiratory syndrome (MERS)-CoV, which can all cause severe lower respiratory tract infections and pose pandemic threats [2,3,4]. The other four CoVs that infect humans (HCoVs)—HCoV-229E, HCoV-NL63, HCoV-OC43 and HCoV-HKU1—primarily cause mild respiratory tract illnesses [1,4]. HCoV-OC43 is believed to have crossed from cattle into humans in the late 19th century, with historical records linking its emergence to the 1890 epidemic known as the “Russian influenza” [3,5]. Today, HCoV-OC43 is a frequent cause of the common cold but can cause severe lower respiratory tract disease in vulnerable or immunocompromised populations [4]. Due to its similar respiratory tract tropism, replication in ciliated cells, and classification as a betacoronavirus, it is commonly used as a Biosafety Level 2 (BSL-2) surrogate for SARS-CoV-2 [2,6,7].

To conduct meaningful studies on CoV biology and pathogen–host interactions, researchers rely on advanced models that reproduce the structural and functional complexity of the human airway. Air–liquid interface (ALI) culture has emerged as the gold-standard in vitro model for the human respiratory tract, as it reproduces key aspects of in vivo airway physiology [8,9,10]. In ALI cultures using primary human bronchial epithelial cells (pHBECs), basal progenitor cells are supplied with nutrient-rich medium from the basal side, while the apical surface is exposed to air. This promotes differentiation into ciliated, goblet, and club cells, which can be identified by characteristic marker genes [8,10,11,12]. Moreover, ALI cultures self-organize into a polarized, pseudostratified epithelium [11], form tight junctions with physiologically relevant transepithelial electrical resistance (TEER) values (>400 Ω·cm^2^) [10,13,14] and exhibit ciliary beating starting from two to three weeks after transition to ALI conditions [9,10,15,16]. A unique feature of ALI cultures is the mucus barrier, which is primarily composed of gel-forming mucins from goblet cells supplemented with immunologically active proteins, for example, from club cells [9,15]. This barrier restricts access of pathogens such as viruses to the epithelium, altering viral entry and replication dynamics and therapeutic outcomes compared with conventional submerged cultures [17,18]. Importantly, ALI systems express host factors that crucially affect viral replication, including entry receptors, innate immune sensors, and ion channels [9,10,17,18,19]. This not only makes ALI cultures highly physiological models but also enables the isolation, propagation and study of viruses that are uncapable of replicating in conventional cell culture systems and immortalized cell lines [20,21].

Although ALI systems are a valuable tool for studying airway biology and respiratory virus pathology, comparability of data is hampered by the lack of standardized procedures for ALI culture. For studies using primary cells in ALI cultures, cell passage numbers between one and six have been employed [2,10,12]. Furthermore, weekly mucus washes are typically initiated after two weeks of ALI culture. However, wash protocols vary considerably among studies, from brief apical rinses with Hank’s Balanced Salt Solution (HBSS) [15] to more intensive mucus removal procedures involving 1 h incubation at 37 °C with rocking [22].

In addition to variables specific for ALI culture, differing viral infection protocols introduce further variation and are often incompletely documented in the literature. HCoV-OC43 infection in pHBEC ALI cultures is most commonly performed apically, with virus diluted in HBSS and inoculation performed for 2 h at 33 °C. After removal of the virus infection mix, three HBSS washes are commonly performed [2,15,23]. Reported variations include inoculation with virus for up to 4 h at 34 °C [24] or infecting in alternative media, such as 1% FCS-DMEM supplemented with antibiotics [25]. Frequently, cells are maintained in minimal medium without heparin for up to 24 h before infection [23] to enhance viral entry, as many viruses including HCoVs use heparan sulfates for host cell attachment [3,9]. Reported replication kinetics in ALI-cultured cells also differ among studies. While some reports indicate that HCoV-OC43 replication peaks 3 to 4 days (d) after infection [2], others observe maximum RNA levels after 4 to 6 d [15,25]. Long-term culture of CoVs, including HCoV-OC43, has been demonstrated for up to 100 d [15] or even 200 d with intermittent re-infections [25]. In contrast, few studies provide details on the timing and frequency of mucus washes prior to infection in ALI culture models. As a key physical and chemical barrier, the extent of mucus removal before infection can significantly modulate susceptibility to viral infection [26,27,28].

Taken together, variations in ALI culture and infection protocols can influence differentiation timelines, reproducibility, and viral replication outcomes [9,12,17]. Given that subtle shifts in epithelial composition or maturation status can alter susceptibility to CoV infection, as shown for SARS-CoV-2, achieving reproducible infection outcomes in ALI cultures requires the careful control and transparent reporting of culture conditions [17].

To enhance the understanding of CoV replication and promote comparable infection outcomes across laboratories, we set out to establish a robust HCoV-OC43 infection model using pHBECs cultured under ALI conditions. We evaluated the impact of seven variables on HCoV-OC43 replication. Instead of applying the classical one-factor-at-a-time (OFAT) methodology, in which only one variable is altered to identify its optimal value while keeping all others constant [29], we employed a Design of Experiments (DoE) approach. In this statistical modeling strategy, several variables are simultaneously varied across specified levels (i.e., specific values assigned to each variable) according to the experimental design. This allows for the study of single-variable as well as interaction effects, providing a more efficient means of experimental optimization. This is of particular importance when complex interdependencies among biological variables are expected [30].

In this work, we reveal the impact of epithelial differentiation and additional variables on HCoV-OC43 replication and identify conditions to achieve robust viral replication in fully differentiated bronchial epithelial ALI cultures.

## 2. Methods

### 2.1. Experimental Design Using the Custom DoE Module in JMP^®^ 18

The effect of seven variables on HCoV-OC43 replication was investigated: (i) passage number of pHBECs, (ii) duration of ALI culture before infection, (iii) timepoint of last mucus wash relative to infection, (iv) number of sequential mucus washes, (v) infection route, (vi) duration of virus inoculation and (vii) interval between infection and harvest. Each variable was classified appropriately in the JMP (SAS Institute Inc., Cary, NC, USA) DoE platform: all variables were defined as continuous variables with the exception of “infection route”, which was defined as a categorical variable. A custom DoE design was employed to analyze linear and quadratic effects of main variables as well as all potential two-way interactions. The number of samples to be included in the experiment was iteratively increased until a high power with a statistical significance of *p* < 0.05 was achieved for all parameters of interest (Supplementary File S3), yielding 120 experimental runs in total (Supplementary File S2). D-, A-, and G-efficiencies were considered for design evaluation (Supplementary Table S3).

### 2.2. Generation of HCoV-OC43 Stock

BHK-21 cells were infected with HCoV-OC43 (sequence available in GenBank under accession number PX101827) at a multiplicity of infection (MOI) of 0.1, followed by incubation at 35 °C and 5% CO_2_. After 4 d, cell culture flasks were shock-frozen at −80 °C to lyse virus-containing cells. After thawing, the cell–virus mixture was transferred to a falcon tube and centrifuged at 3000× *g* for 10 minutes (min) to remove cell debris. The supernatant containing virus was aliquoted and stored at −80 °C.

To determine viral infectivity, a tissue culture infectious dose 50 (TCID_50_) assay was performed. Briefly, BHK-21 cells were infected with serial dilutions of HCoV-OC43 and returned to the incubator (35 °C, 5% CO_2_). The cytopathic effect (CPE) were monitored microscopically each day. On d4 and d5, a clear CPE was visible. The TCID_50_ value was calculated on d5 using the Spearman–Kärber method. The viral stock titer corresponded to 1.3 × 10^8^ plaque-forming units (PFU)/mL.

### 2.3. ALI Culture of pHBECs

ALI experiments were conducted using pHBECs acquired from Lonza (Basel, Switzerland; cat. No. CC-2540S). Passages 1 or 3 of the same donor were used for infection experiments using the DoE approach (batch number: 23TL023428). Passage 2 of a different donor (batch number: 22TL108516) was used for validation experiments investigating the influence of ALI culture duration and infection route on viral replication. Under submerged culture conditions, pHBECs were cultured in PneumaCult™ Ex-Plus medium (STEMCELL Technologies, Vancouver, BC, Canada; cat. No. 05040) with Hydrocortisone (STEMCELL Technologies; cat. No. 07925) until a confluency of 70–80% was reached. Cells were detached from the flask with Accutase (CLS Cell Lines Services GmbH, Eppelheim, Germany; cat. No. 830100), centrifuged for 5 min at 300× *g*, and seeded into Transwell^®^ inserts (Corning, NY, USA; cat. No. 3480) coated with collagen IV (Merck KGaA, Darmstadt, Germany; cat. No. 7521). A total of 50,000 cells/insert were seeded in PneumaCult™-Ex Plus complete medium, and 3 d after seeding, the medium on the apical side of cells was removed. Cells were supplied with PneumaCult™-ALI maintenance medium (STEMCELL Technologies; cat. No. 05001) with Hydrocortisone and Heparin (STEMCELL Technologies; cat. No. 07980) from the basal side, with medium changes performed every 2–3 d.

For mucus washes, 200 µL of HBSS (Gibco, Thermo Fisher Scientific, Waltham, MA, USA; cat. No. 14185052) was applied to the apical surface of the cells and incubated for 7 min at 37 °C. The liquid was then gently pipetted up and down five times before removal. For ALI cultures maintained until d28, three consecutive mucus washes were performed on d14 to remove accumulated mucus.

In validation experiments using cells from a different donor, three consecutive mucus washes (7 min each) were performed weekly as well as on the day of infection.

### 2.4. TEER Measurements

TEER measurements were performed weekly from d7 to d28 of culture at the ALI to assess epithelial barrier integrity in uninfected ALI cultures. Measurements were conducted using an STX electrode connected to an EVOM2 voltohmmeter (World Precision Instruments, Sarasota, FL, USA) according to the manufacturer’s instructions. Values were recorded in ohms (Ω) and normalized to the surface area of the ALI insert to obtain values in Ω·cm^2^.

### 2.5. Lactate-Dehydrogenase (LDH) Release Assay

Basal media from pHBECs cultured for 0–28 d under ALI conditions were collected weekly (*n* = 2) and stored at −80 °C until analysis. LDH release into basal media of ALI cultures was measured using the CyQUANT™ LDH and G6PD Cytotoxicity Assay Kit (Invitrogen, Thermo Fisher Scientific; cat. No. C20303) according to the manufacturer’s instructions. Fluorescence intensity was measured on a microplate reader at Ex/Em 546/590 nm, and the LDH positive control provided in the kit was used as a control. The Kruskal–Wallis test with Dunn’s multiple comparisons test was performed to identify significant increases in LDH activity of each group compared with d7 of culture under ALI conditions.

### 2.6. Infection with HCoV-OC43

pHBECs were infected with HCoV-OC43 via the apical or basal side of the transwell with 1.6 × 10^5^ PFU/mL in 200 µL. After the designated time of incubation with virus at 35 °C and 5% CO_2_ according to the experimental design matrix (Supplementary Figure S2), the infection mix was aspirated, cells were carefully washed 3× with 200 µL of phosphate-buffered saline (PBS) either on the apical or basal side, and the ALI was restored. Cells were incubated at 35 °C and 5% CO_2_ for the designated number of days until harvest of nucleic acid was performed according to the DoE design matrix.

In validation experiments using cells from a different donor, cells were inoculated with 1.6 × 10^5^ or 1.6 × 10^4^ PFU/mL for 3 h before removal and washing 3× with PBS. Harvest of nucleic acid was performed 3 d after infection.

### 2.7. RNA Extraction and RT-qPCR

Viral RNA was quantified from apical, intracellular and basal compartments. For this, apical washes of ALI inserts were obtained by performing three consecutive apical washes with a total of 400 µL of PBS (Sigma-Aldrich, St. Louis, MO, USA; cat. No. D8537). PBS was pipetted up and down 5×, and the three washes were combined for nucleic acid extraction. A volume of 400 µL of basal medium was collected from basal compartments. For the harvest of cell lysates, cells were incubated with a 0.5% trypsin–EDTA solution (Gibco, Thermo Fisher Scientific; cat. No. 15400054) for 15 min at 35 °C, after which the detached cells were obtained through vigorous pipetting and the careful scratching of ALI inserts. A MagNA Pure 96 Instrument (Roche, Basel, Switzerland) was employed to extract nucleic acids from apical, intracellular and basal compartments (program: PathogenUniversal 200) using the MagNA Pure 96 DNA and Viral NA Small Volume kit (Roche; cat. No. 06543588001). For RT-qPCR, Taqman™ Fast Virus 1-Step Mastermix (Applied Biosystems, Warrington, UK; cat. No. 4444432) was used. PCR was performed with the ViiA7 Real-time PCR system (Life Technologies, Foster City, CA, USA). Absolute quantification was achieved using an RNA standard containing the PCR target sites. In validation experiments using cells from a different donor, viral RNA harvest was performed using RA1 buffer (Macherey-Nagel, Düren, Germany; cat. No. 740961) + 20 mM DTT (Merck KGaA; cat. No. 10197777001).

For RT-qPCR of ALI differentiation markers, uninfected ALI samples were collected on d0, d7, d14, d21 and d28 of ALI culture (*n* = 2). For this, RA1 buffer + 20 mM DTT was pipetted apically onto pHBECs, followed by nucleic acid extraction and RT-qPCR with the Taqman™ Fast Virus 1-Step Mastermix. Primers, probes and PCR cycling conditions are provided in Supplementary Tables S4 and S5.

### 2.8. Data Processing and Statistical Modeling in JMP^®^ 18

Data distribution was assessed using histograms, normal quantile plots, and the Shapiro–Wilk test in JMP. To improve normality and model fit, viral genomic copy numbers quantified by RT-qPCR were log_10_-transformed (Supplementary Figure S1). For data analysis, a least-squares regression model was employed [31,32,33]. All variables were included as linear and quadratic terms, and all possible interaction terms were considered. Infection route was included as a categorical variable, while the other six variables were defined as continuous. This modeling approach corresponds to a multivariable regression framework with response surface methodology to account for non-linear and interaction effects. Model performance was assessed using the coefficient of determination (R^2^), adjusted R^2^, root mean square error (RMSE), and lack-of-fit testing. Model assumptions, including normality and homoscedasticity of residuals, were assessed by visual inspection of residual plots (Supplementary Figure S1).

The statistical significance of individual model terms was evaluated using *p*-values from the effect summary panel within the multivariable regression model of JMP. These *p*-values indicate whether a given variable or interaction term significantly contributes to variation in the y-variable. Due to the reduced factorial design and the treatment of most variables as continuous predictors, the model does not allow for direct comparison between individual variable levels.

To visualize the effect of individual variables on HCoV-OC43 replication, the integrated prediction profiler tool was used. For regression plots not displaying “duration of ALI culture before infection,” this variable was fixed at d21 to visualize the impact of the other variables specifically in the context of fully differentiated cultures. Desirability functions (range of 0–1) were used to identify variable settings that maximize predicted HCoV-OC43 copy number when infecting on d21 of ALI culture for all remaining variables. The predicted optimal parameter settings from the prediction profiler tool are summarized in Table 1. Two-dimensional plots in this manuscript represent the effect of a single variable on HCoV-OC43 gRNA quantity while all other variables are held constant at their maximal values.

Interaction effects between two variables were visualized using interaction profiles derived from the model. In these plots, non-displayed variables were held at the same maximum settings as for single-variable plots, ensuring consistency between single-factor and interaction visualizations.

## 3. Results

### 3.1. pHBECs Reach Full Maturity After 21 d of Culturing at the ALI

**Figure 1:**
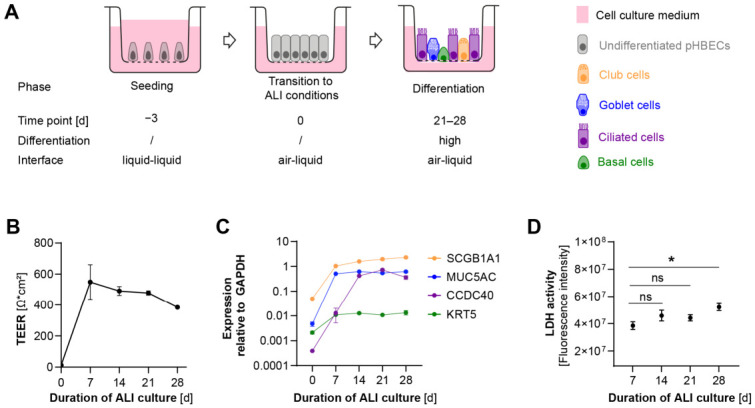
Maturation and differentiation of primary human bronchial epithelial cells (pHBECs) cultured at the air–liquid interface (ALI). (**A**) Schematic representation of the ALI model used in this study. pHBECs were cultured at the ALI for up to 28 d. (**B**) Epithelial barrier integrity was assessed by weekly monitoring transepithelial electrical resistance (TEER) values. (**C**) Cellular differentiation was monitored through reverse transcription quantitative PCR (RT-qPCR) for cell-specific markers, including secretoglobin family 1A member 1 (SCGB1A1) for club cells, mucin 5AC (MUC5AC) for goblet cells, coiled-coil domain-containing protein 40 (CCDC40) for ciliated cells and keratin 5 (KRT5) for basal cells. mRNA expression levels were normalized to glyceraldehyde-3-phosphate dehydrogenase (GAPDH). (**D**) Cytotoxicity was assessed by measurements of lactate dehydrogenase (LDH) activity in basal media. The Kruskal–Wallis test with Dunn’s multiple comparisons test was performed to identify significant increases in LDH activity compared with d7 of culture at the ALI. Mean +/− SD is shown. ns = not significant; * *p* < 0.05.

To establish and characterize the ALI culture model used throughout this study, pHBECs were first seeded onto transwells. After 3 d, ALI conditions were established by removing media from the apical compartment (Figure 1A) and replacing expansion medium with ALI maintenance medium in the basal compartment. Cells were cultured for up to 28 d, and epithelial maturation and cellular differentiation status were monitored. Weekly TEER measurements revealed a sharp rise in barrier integrity within the first week of ALI establishment, with values peaking over 500 Ω·cm^2^ on d7 (Figure 1B). TEER values remained stable above 450 Ω·cm^2^ for 21 d, before slightly declining in the fourth week of culture. Cellular differentiation was monitored by quantifying mRNA of genes specific for the cell types of the human bronchial epithelium by RT-qPCR. These included secretoglobin family 1A member 1 (SCGB1A1) for club cells [34,35], mucin 5AC (MUC5AC) for goblet cells [36,37], keratin 5 (KRT5) for basal cells [38,39], and coiled-coil domain-containing protein 40 (CCDC40) for ciliated cells [40]. Increases in expression of all four marker genes during the first week of ALI culture indicated differentiation into club, goblet, basal and ciliated cells (Figure 1C). MUC5AC expression peaked on d14 and then stabilized, consistently with microscopical assessment of mucus secretion. KRT5 mRNA levels reached an early peak on d7 and then remained stable until d28, while expression of CCDC40 mRNA peaked on d21. SCGB1A1 mRNA levels steadily increased until d28 [26]. Quantification of LDH activity in the culture medium indicated high overall cell viability, with a slight increase in cytotoxicity observed on d28 compared with d7 after culture at the ALI (Figure 1D). Overall, pHBECs cultured at the ALI recapitulated key features of the human airway epithelium, including differentiation into major epithelial cell types and barrier formation. This was supported by robust expression of marker genes for club, goblet, basal, and ciliated cells, together with tight junction formation, as indicated by increasing TEER values. After 21 d of culture, ALI cultures exhibited both high maturation and viability.

### 3.2. DoE Algorithm Generates 120 Unique Experimental Conditions

In our study we investigated the impact of seven variables on HCoV-OC43 replication, including (i) the passage number of pHBECs, (ii) the duration of ALI culture before infection, (iii) the timepoint of last mucus wash relative to infection, (iv) the number of sequential mucus washes, (v) the infection route, (vi) the duration of virus inoculation, and (vii) the interval between infection and harvest.

**Figure 2:**
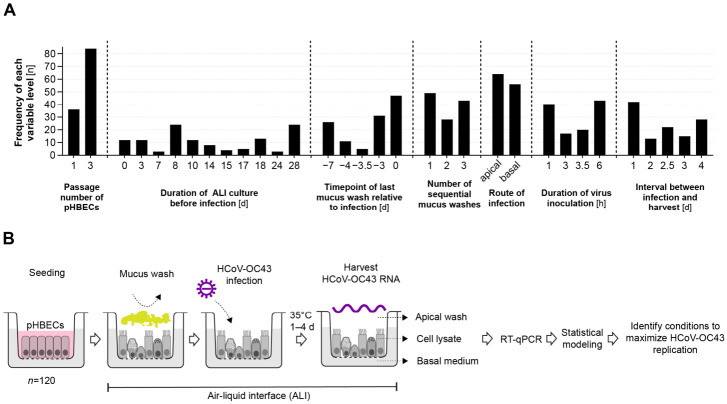
Design of Experiments (DoE) algorithm generates 120 experimental conditions to assess the effect of seven variables on HCoV-OC43 replication. (**A**) A reduced factorial custom design matrix was generated such that the effect of the individual variables and interaction effect of several variables on viral gRNA quantities could be investigated with high statistical power, resulting in 120 unique experimental conditions. The frequency of each variable level in the design is shown. (**B**) General workflow used to assess HCoV-OC43 replication in 120 ALI inserts is depicted. Colour code: pink, culture medium; yellow, mucus; purple, virus/viral RNA.

Due to the high number of variables and variable levels (i.e., the specific conditions tested for each variable) inherent in our study design, as well as the resource-intensive nature of ALI cultures, we adopted a reduced factorial rather than a full factorial DoE approach [30,41]. Consequently, only a subset of all possible variable level combinations was included in our experimental design. Variable levels were either defined manually or generated by the DoE algorithm in JMP, resulting in two-to-eleven levels per variable (Figure 2A). The number of samples was increased until a statistical power of 1.0 at a significance level of *p* = 0.05 was achieved for all variables and variable interactions, yielding 120 unique experimental conditions (Supplementary Files S2 and S3). The DoE algorithm does not distribute the frequency of tested variable levels equally but preferentially weights the lowest and highest tested levels of each variable.

After transition to ALI conditions, pHBECs were subjected to the 120 different experimental conditions according to the design matrix. The general workflow involved mucus washes before infection, removal of viral inoculum and washing (Figure 2B). At the harvest timepoint, HCoV-OC43 gRNA was quantified from apical, basal and intracellular compartments by RT-qPCR as a surrogate marker for viral replication. To identify key determinants of HCoV-OC43 replication in ALI cultures, we employed a least-squares fit regression model [31,32,33], considering all linear, quadratic, and interaction terms. Conditions that maximize HCoV-OC43 replication in fully mature pHBECs cultured at the ALI were identified using the prediction profiler tool within the JMP model.

### 3.3. HCoV-OC43 Replication Can Be Monitored by Quantifying Viral RNA from Cell Lysate or Apical Washes

**Figure 3:**
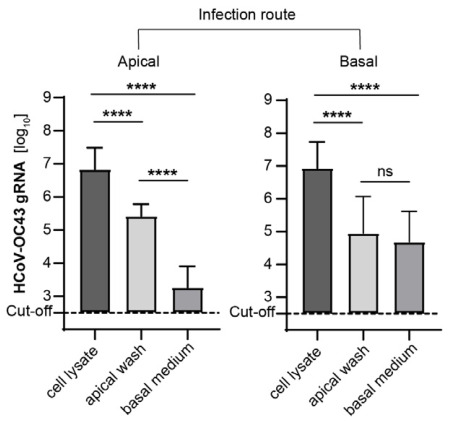
Evaluation of different sample types to assess HCoV-OC43 replication in ALI-cultured pHBECs. pHBECs cultured at the ALI were subjected to 120 experimental conditions according to our reduced factorial custom DoE. Viral genomic RNA (gRNA) was quantified from cell lysates (*n* = 120), apical washes (*n* = 119) and basal media (*n* = 120) by RT-qPCR. Values below the detection limit of RT-qPCR were set to the cut-off (log_10_ = 2.5), indicated by a dashed line. Data are presented as median with interquartile range. Statistical differences were evaluated using the Kruskal–Wallis test with Dunn’s multiple comparisons test. ns = not significant; **** *p* < 0.0001.

HCoV-OC43 gRNA was quantified by RT-qPCR across three ALI compartments: basal media, cell lysates, and apical washes. Viral RNA was detected in all cell lysate samples, whereas only 111 of 119 apical wash samples and 89 of 120 basal medium samples tested positive (Supplementary File S2). Irrespective of the infection route, the highest viral gRNA levels were detected in cell lysates, with lower viral RNA observed in apical washes (Figure 3). Notably, all eight apical wash samples with undetectable viral RNA originated from cultures infected via the basal route, suggesting failure of productive infection in these cases.

In basal media, viral RNA levels differed substantially depending on the route of infection. For infection via the apical side, gRNA was significantly lower than in the other two sample compartments, with the median value close to the PCR cut-off. Notably, 29 of the 31 basal medium samples with undetectable viral gRNA were derived from apically infected cultures, representing nearly half of all apically infected samples (*n* = 64) and contributing to the low median gRNA observed for the apical infection route.

Overall, the distribution of viral gRNA indicated enrichment within cell lysates, consistent with intracellular viral replication, with lower levels of viral RNA detected in apical washes. In basal media, only around half of the apically infected samples were RT-qPCR-positive at the harvest timepoint (days 1–4 post-infection), suggesting that detected RNA largely represented residual inoculum from basal infection that was not completely removed during post-infection washing. Furthermore, these findings suggest that release of mature virions occurred predominantly from the apical rather than the basal side of the epithelium. Therefore, we focused all subsequent analyses on datasets derived from cell lysate and apical wash compartments and did not further consider basal medium samples.

### 3.4. HCoV-OC43 gRNA Peaks After 3 d in Cell Lysates and After 4 d in Apical Washes

**Figure 4:**
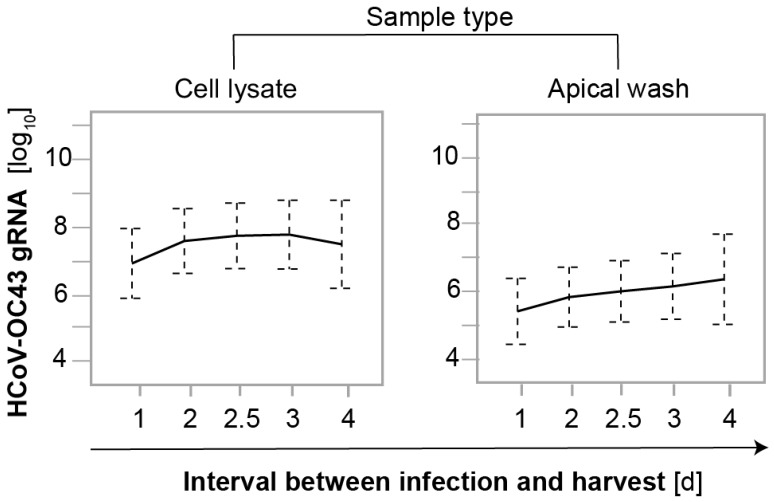
Replication kinetics of HCoV-OC43 in pHBECs cultured at the ALI across two different compartments. pHBECs cultured at the ALI were subjected to 120 experimental conditions according to our reduced factorial custom DoE. Viral gRNA was quantified from cell lysates (*n* = 120) and apical washes (*n* = 119) by RT-qPCR. Values below the detection limit of RT-qPCR were set to cut-off (log_10_ = 2.5). A least-squares fit regression model including all linear, quadratic, and interaction terms was used to assess HCoV-OC43 replication kinetics in different sample compartments. The plot shows the predicted response curve, with all other variables fixed at the levels yielding maximum viral gRNA levels when infecting on d21 of ALI culture. Dashed lines indicate 95% confidence intervals.

Using a multivariable regression model to evaluate data from our DoE, we investigated viral replication kinetics across two ALI compartments. A significant effect of the interval between infection and harvest on viral gRNA levels was observed in both cell lysates and apical washes (Supplementary Tables S1 and S2). HCoV-OC43 gRNA levels were found to increase over time, reaching peak values 3 d after infection in cell lysates and after 4 d in apical washes (Figure 4), most likely reflecting a delayed release of viral particles into the extracellular compartment. The consistent increase in viral gRNA levels over time indicated active viral replication.

### 3.5. Maturation of the Respiratory Epithelium Negatively Impacts Apical HCoV-OC43 Infection

**Figure 5:**
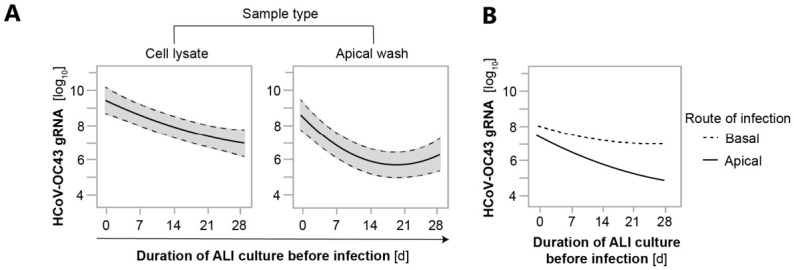
Effect of ALI culture duration on HCoV-OC43 gRNA quantities following basal or apical infection. pHBECs cultured at the ALI were subjected to 120 experimental conditions according to our reduced factorial custom DoE. pHBECs were infected at different timepoints between 0 and 28 d of ALI culture via either the apical or basal route. A least-squares fit regression model including all linear, quadratic, and interaction terms was used to assess the effect of ALI culture duration and infection route on HCoV-OC43 gRNA levels. Values below the detection limit of RT-qPCR were set to the cut-off (log_10_ = 2.5). (**A**) Effect of ALI culture duration on viral gRNA levels. (**B**) Interaction effect of ALI culture duration and infection route on viral gRNA levels for cell lysate samples. Shaded areas indicate 95% confidence intervals. Sample sizes: *n* = 120 (cell lysates) and *n* = 119 (apical washes).

To evaluate how the differentiation of pHBECs at the ALI affected HCoV-OC43 replication, we evaluated HCoV-OC43 gRNA levels in relation to ALI culture duration prior to infection using our multivariable regression model. This revealed a significant effect of ALI culture duration on viral RNA quantities in both cell lysates (Supplementary Table S1) and apical washes (Supplementary Table S2). Viral gRNA levels were the highest when infection was performed at initiation of ALI culture (d0) and progressively declined with the increase in epithelial maturation at the ALI (Figure 5A).

Furthermore, the regression model indicated that the effect of ALI culture duration was dependent on the infection route (Supplementary Table S1). Viral gRNA levels were more dependent on ALI culture duration following apical rather than basal infection (Figure 5B). Overall, the model predicted higher HCoV-OC43 RNA when infecting through the basal medium, especially for fully differentiated ALI cultures.

### 3.6. Repetitive Mucus Washes Enhance HCoV-OC43 Infection and Replication in Fully Differentiated pHBECs Cultured at ALI

**Figure 6:**
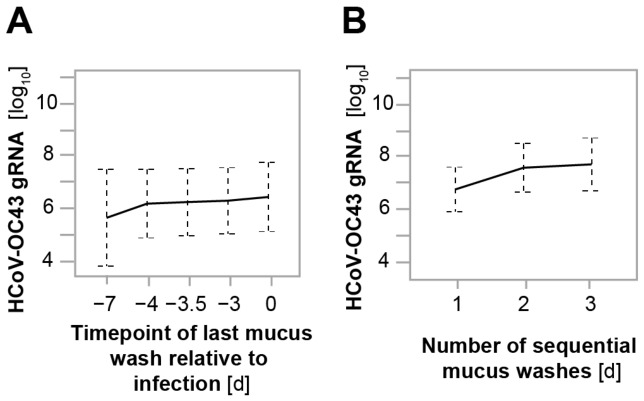
Effect of mucus wash timing and frequency on HCoV-OC43 gRNA levels in ALI-cultured pHBECs. pHBECs cultured at the ALI were subjected to 120 experimental conditions according to our reduced factorial DoE. Before infection, mucus was removed through one, two or three sequential apical washes at different timepoints, ranging from the same day to 7 d prior to infection. A least-squares fit regression model including all linear, quadratic, and interaction terms was used to assess the effect of mucus wash timepoint and frequency on HCoV-OC43 gRNA levels. Values below the detection limit of RT-qPCR were set to the cut-off (log_10_ = 2.5). (**A**) Effect of the timepoint and (**B**) number of sequential mucus washes on viral gRNA levels, shown for apical wash and cell lysate samples, respectively. The plot shows the predicted response curve, with all other variables fixed at the levels yielding maximum viral gRNA levels when infecting on d21 of ALI culture. Dashed lines indicate 95% confidence intervals. Sample sizes: *n* = 120 (cell lysates) and *n* = 119 (apical washes).

To assess whether the mucus barrier limits HCoV-OC43 infection of ALI-cultured pHBECs, we investigated how the removal of mucus through apical washes would affect endpoint viral gRNA levels. For this, either one, two or three apical washes were performed at different timepoints ranging from the same day to 7 d prior to infection, according to the experimental design matrix. HCoV-OC43 gRNA from different sample compartments was once more evaluated within our multivariable regression model, which did not indicate a significant effect of the timepoint of the last mucus wash (Figure 6A). In contrast, the number of sequential mucus washes was identified as a significant parameter influencing HCoV-OC43 gRNA quantities in cell lysate samples (Supplementary Table S1). Regression plots indicated higher endpoint gRNA levels following two or three apical washes prior to infection compared with a single wash (Figure 6B). In summary, repetitive apical washes prior to infection increased endpoint HCoV-OC43 gRNA levels, potentially by facilitating cellular infection and viral spread.

### 3.7. pHBECs at Passage 3 Support HCoV-OC43 Replication at Levels Comparable to Passage 1 in Fully Differentiated ALI Cultures

**Figure 7:**
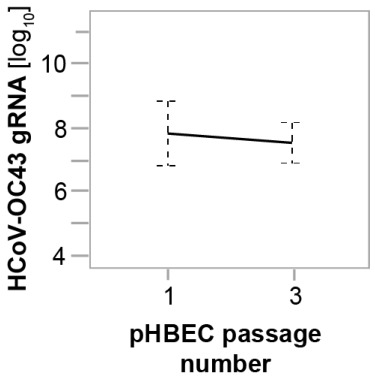
Effect of pHBEC passage number on HCoV-OC43 replication in ALI cultures. pHBECs cultured at the ALI were subjected to 120 experimental conditions according to our reduced factorial custom DoE. A least-squares fit regression model including all linear, quadratic, and interaction terms was used to assess the effect of pHBEC passage number on HCoV-OC43 gRNA levels and is shown for cell lysate samples. The plot shows the predicted response curve, with all other variables fixed at the levels yielding maximum viral gRNA levels when infecting on d21 of ALI culture. Values below the detection limit of RT-qPCR were set to the cut-off (log_10_ = 2.5). Dashed lines indicate 95% confidence intervals. Sample size: *n* = 120 (cell lysates).

To evaluate whether the cell passage number affected HCoV-OC43 replication, pHBECs at passages one and three derived from the same donor were included in the experimental design. HCoV-OC43 gRNA from cell lysates and apical wash samples was evaluated within our multivariable regression model, which indicated a slight trend of higher endpoint gRNA when using passage 1 over passage 3 cells (Figure 7); however, this effect was not significant. These data indicate that passage 3 pHBECs support robust HCoV-OC43 replication in fully differentiated ALI cultures, with gRNA levels comparable to those obtained when infecting passage 1 cells.

### 3.8. The Duration of Virus Inoculation Has No Effect on HCoV-OC43 gRNA Levels in pHBEC ALI Cultures

**Figure 8:**
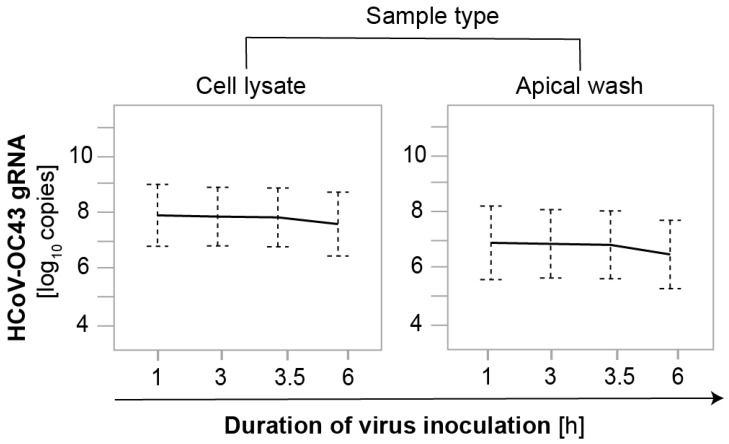
Effect of viral inoculation duration on endpoint HCoV-OC43 gRNA levels. pHBECs cultured at the ALI were subjected to 120 experimental conditions according to our reduced factorial custom DoE. pHBECs were infected with HCoV-OC43 for 1 to 6 h. A least-squares fit regression model including all linear, quadratic, and interaction terms was used to assess the effect of viral inoculation duration on HCoV-OC43 gRNA levels. The plot shows the predicted response curve, with all other variables fixed at the levels yielding maximum viral gRNA levels when infecting on d21 of ALI culture. Values below the detection limit of RT-qPCR were set to the cut-off (log_10_ = 2.5). Dashed lines indicate 95% confidence intervals. Sample sizes: *n* = 120 (cell lysates) and *n* = 119 (apical washes).

To examine how the duration of virus inoculation would affect HCoV-OC43 replication, pHBECs were exposed to virions for periods ranging from 1 to 6 h. Multivariable regression analysis of the DoE data revealed no significant effect of this variable on viral gRNA (Figure 8), suggesting that 1 h of viral exposure was sufficient to support robust viral propagation in our study.

### 3.9. Validation of Key Findings Using pHBECs from Another Donor

Due to potential donor-to-donor variability in pHBECs and the exploratory nature of the DoE approach, we performed a validation experiment using an independent donor and biological replicates to confirm key results obtained using the DoE approach. For this, we focused on investigating the effect of ALI culture differentiation and infection route on HCoV-OC43 replication 3 d post-infection, given that these variables demonstrated a significant impact on viral gRNA quantities according to our multivariable regression model. To this end, pHBECs derived from another donor were infected with HCoV-OC43 via the apical or the basal route at different timepoints after initiation of ALI culture. In addition to quantifying viral gRNA, total viral RNA was assessed by RT-qPCR targeting the nucleocapsid (NC)-encoding sequence, which is present in all CoV-derived transcripts [42].

**Figure 9:**
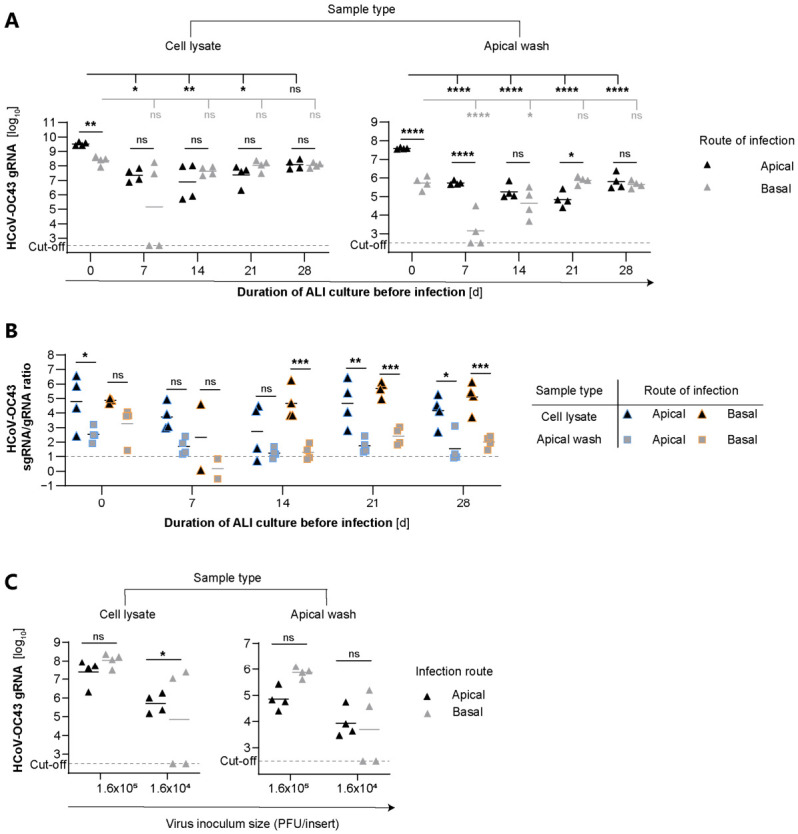
Effect of ALI culture duration, infection route and virus dose on HCoV-OC43 replication in pHBECs from a second donor. pHBECs from another donor were cultured at ALI for 0–28 d and infected for 3 h via the apical or basal route. Viral RNA was harvested 3 d post-infection. Viral gRNA and total RNA were quantified by RT-qPCR in cell lysate and apical wash compartments. (**A**) The effect of ALI culture duration and infection route on HCoV-OC43 gRNA quantities are shown. (**B**) The ratio of HCoV-OC43 subgenomic (sg) to gRNA for different infection routes and sample compartments is shown. In the absence of sgRNA-derived signal from active replication, the sgRNA:gRNA ratio is approximately one, as indicated by the dashed line. (**C**) pHBECs were cultured at the ALI for 21 d and were then infected with HCoV-OC43 at virus inoculum sizes of 1.6 × 10^5^ or 1.6 × 10^4^ PFU via the apical or basal route for 3 h. Viral RNA harvests were performed 3 d post-infection. (**A**,**C**) Values below the detection limit of RT-qPCR were set to the cut-off (log_10_ = 2.5), indicated by a dashed line. (**A**–**C**) Data are presented as means (*n* = 4). Statistical analyses were performed using two-way ANOVAs followed by multiple comparisons tests. Šídák’s test was used in (**A**) to assess differences between infection routes at each timepoint, in (**B**) to evaluate differences in sgRNA:gRNA ratios between sample compartments and in (**C**) to assess differences between infection route for each viral dose. Dunnett’s test was used in (**A**) to compare each infection timepoint to d0. ns = not significant; * *p* < 0.05; ** *p* < 0.01; *** *p* < 0.001; **** *p* < 0.0001.

For experiments using an alternate donor, the highest levels of viral RNA were measured following apical infection in undifferentiated cells that were infected on the same day that ALI culture was initiated (d0), as demonstrated by both viral gRNA (Figure 9A) and total RNA (Supplementary Figure S2A) quantified from apical washes and cell lysates. While significantly lower viral RNA was observed following basal infection compared with apical infection in undifferentiated cells, the impact of cellular differentiation on viral replication appeared less pronounced. Nonetheless, significantly lower viral RNA was detected in apical washes on d7 and d14 in cells infected via the basal route, whereas gRNA levels reached values comparable to those observed on d0 at later timepoints (d21 and d28). Viral RNA was not detected in two of four replicates following basal infection on d7 of ALI culture, suggesting that basal infection may result in less consistent replication outcomes (Figure 9A).

Overall, experiments using cells from a different donor confirmed the trends from our DoE-based approach, as higher gRNA levels were observed in both experiments following apical infection in undifferentiated pHBECs compared with later stages of differentiation. Furthermore, data from both experiments suggest a stronger dependence of endpoint viral RNA on ALI culture differentiation following apical infection, whereas basal infection was less affected by epithelial differentiation. The validation experiment with the alternate donor further indicated that endpoint gRNA levels following infection of pHBECs cultured for 21 and 28 d at the ALI were comparable to those observed on day 0.

To further evaluate HCoV-OC43 replication, we additionally calculated the ratio of subgenomic (sg) RNA to gRNA based on RT-qPCR signals from the NC and RNA-dependent RNA polymerase (RdRP) targets. RT-qPCR targeting the NC region detects both gRNA and all sgRNAs, thereby reflecting total viral RNA. sgRNAs are abundantly produced during active replication but are not packaged into virions. Therefore, during replication, the relative contribution of sgRNA-derived signal increases, resulting in elevated sgRNA:gRNA ratios, which can be interpreted as an indicator of ongoing viral replication [42]. In line with this, sgRNA:gRNA ratios were generally higher in cell lysates than in apical washes (Figure 9B). In apical wash samples, sgRNA:gRNA ratios generally remained low and were frequently close to one, indicating that this sample type primarily contained mature virions released into the extracellular compartment.

In addition, we expanded the validation experiment to investigate the effect of virus inoculum size during infection. To this end, pHBECs were cultured at the ALI for 21 d and were infected with either 1.6 × 10^5^ PFU/insert, corresponding to the virus dose that was used for all previous experiments, or a 10-fold lower dose of 1.6 × 10^4^ PFU/insert. While viral RNA was detected in all four replicates at the higher dose for both apical and basal infection, only two of four replicates exhibited detectable gRNA at the lower dose following basal infection (Figure 9C). This resulted in slightly lower mean HCoV-OC43 gRNA levels at the lower inoculum in basal infection compared with apical infection. Similar results were obtained for total HCoV-OC43 RNA (Supplementary Figure S2B).

Collectively, using pHBECs from an independent donor and a conservative experimental design with four biological replicates and alternative statistical analyses, as well as additional quantification of total viral RNA and sgRNA, we were able to confirm the key findings of our DoE experiment. Furthermore, we demonstrate that increasing the viral inoculum size may improve the robustness of basal infection.

## 4. Discussion

ALI cultures reproduce key morphological and functional features of the human airway epithelium, making them superior to conventional submerged models for studying respiratory viruses. However, protocols for viral infection in ALI cultures remain poorly standardized across laboratories. Important experimental parameters, including mucus washing or inoculation duration with virus, are often insufficiently reported or evaluated. This lack of standardization contributes to variability in infection outcomes and hampers reproducibility across laboratories. A key strength of this study is the application of a reduced factorial DoE methodology to systematically explore multiple variables and their interactions while reducing experimental runs, thereby enabling identification of key drivers of HCoV-OC43 replication in bronchial epithelial ALI cultures. Here, we identify epithelial differentiation as a key determinant of HCoV-OC43 replication in primary bronchial epithelial ALI cultures and identify experimental parameters that promote robust viral replication in fully mature cultures using multivariable regression analysis.

In our study, HCoV-OC43 gRNA peaked on d3 in cell lysates and d4 in apical washes, consistent with previous studies reporting peak HCoV-OC43 replication between 3 and 6 d after infection [2,15,25,43]. However, because replication was monitored only through d4 post-infection, the full replication peak in the apical compartment may not have been captured. Viral replication could not be reliably quantified in basal medium, as indicated by the high number of PCR-negative samples following apical infection. This is likely due to the predominantly apical release of progeny HCoV-OC43 virion [2] and residual viral RNA from the inoculum. We therefore conclude that apical wash and cell lysate, but not basal medium samples, provide reliable readouts of HCoV-OC43 replication in ALI cultures.

By recapitulating the differentiated airway epithelium, the ALI model supports replication of respiratory viruses that fail to grow in submerged cultures or immortalized cell lines, including human rhinovirus C, HCoV-HKU1, and various primary clinical isolates [2,9,20,21]. For most CoVs, including HCoV-OC43, infection is reported to be most efficient in ciliated cells [2]. Consistent with this, Thaler et al. showed that greater ciliation of pHBECs at week 5 versus week 3 after ALI culture correlated with increased susceptibility to SARS-CoV-2 infection [17]. However, other studies have shown that HCoV-OC43 and other respiratory viruses such as RSV also replicate efficiently in immortalized extrapulmonary cell lines under conventional submerged culture conditions [7,9,44,45,46]. In our study, the duration of ALI culture emerged as the strongest determinant of HCoV-OC43 gRNA levels, with the highest viral RNA quantities observed following infection of undifferentiated pHBECs and a progressive decrease in more differentiated ALI cultures, particularly following infection via the apical route. This finding was reproducible across both the DoE-based screening experiment and the independent validation experiment using a second donor.

This observation could be explained by basal cell differentiation into specialized epithelial cell types, together with the development of a pseudostratified epithelial architecture and the formation of tight junctions, which likely reduce viral access to susceptible cells and could thereby potentially limit both infection and replication. In addition, the high replication competence of HCoV-OC43 in less mature ALI cultures may be facilitated by the absence of mucociliary and innate immune defenses [9]. This interpretation is supported by our observation that repeated mucus washes significantly increased endpoint viral gRNA levels and that infection through the basal route was less dependent on ALI culture differentiation than infection through the apical route. Another possible explanation for the lower viral RNA levels observed in more differentiated ALI cultures is potential lower cellular viability following prolonged ALI culture. Overall, the differential replication of HCoV-OC43 in immature versus fully differentiated bronchial epithelial ALI cultures highlights important factors governing susceptibility to infection and viral replication, suggesting avenues for further mechanistic investigation.

Mucus serves as a major physical and biochemical barrier that restricts pathogen access to the respiratory epithelium and may therefore substantially influence infection dynamics [15]. More rigorous mucus removal protocols, including extended washing with agitation [22] or the use of mucolytic agents such as DTT, have previously been reported [47,48] and may further improve infection efficiency. Notably, apical infection of fully differentiated pHBECs cultured at the ALI with a ten-fold lower viral inoculum still yielded productive infection in our study, suggesting that the applied washing protocol (three mucus washes, 7 min each, on the day of infection) either removed mucus very efficiently or that the lower dose used for infection still contained a sufficient amount of infectious particles to initiate viral replication.

While infection of cells from the apical side reflects the natural route of viral entry into the respiratory epithelium, standard protocols typically involve applying liquid inoculum to the apical surface, which transiently disrupts the ALI. Notably, even short-term liquid exposure has been shown to induce significant transcriptional and functional changes in ALI cultures [18]. Although typical inoculation periods for HCoV-OC43 are only 2–3 h [2,15,23], the potential impact of ALI disruption is rarely considered. Reducing the duration of apical infection to 1 h may help preserve epithelial integrity. Our findings support this, as varying inoculation times between 1 and 6 h did not affect viral replication. As an alternative infection route preserving ALI conditions, we evaluated infection of pHBECs via the basal route. Upon infection of pHBECs grown under ALI conditions for 21 d within our validation experiment using a different donor, basal infection resulted in slightly higher peak HCoV-OC43 gRNA levels compared with apical infection. However, apical infection yielded more consistent replication throughout all infection timepoints, whereas infection via the basal route showed greater variability in viral copy numbers, with failed replication observed in two of four replicates following infection on d7 of ALI culture with the second donor. In addition, successful viral propagation via the basal route was dependent on virus inoculum size, with a ten-fold lower virus dose resulting in detectable viral RNA in only 50% of replicates.

Overall, our data suggest that basal infection may represent an alternative route of viral entry when aerosol-based apical infection is not feasible, although further optimization is required to achieve more consistent infection and replication. For viral attachment and entry, HCoV-OC43 engages 9-O-acetylated α2,8-linked disialosides, HLA class I molecules, and potentially additional, so-far unidentified receptors [45,46,49,50,51]. The expression of these receptors is not restricted to ciliated cells but is more broadly distributed across the respiratory epithelium [52,53], which may confer susceptibility to HCoV-OC43 infection at early stages of epithelial differentiation as well as via the basal side. Furthermore, a preference for undifferentiated over differentiated pHBECs has been described for other respiratory viruses. For example, Chan et al. reported reduced replication of the avian H5N1 influenza strain in well-differentiated compared with undifferentiated ALI cultures [16]. Future studies should further investigate viral entry mechanisms, cell-type tropism, and host responses under basal infection conditions, as well as technical factors such as insert pore size and density, which may influence infection efficiency via the basal compartment.

With respect to the different pHBEC passages used, our data indicate only a small advantage of using passage 1 over passage 3 cells when infecting fully mature ALI cultures. Previous studies have similarly reported that pHBECs between passages 1 and 3 are capable of producing well-differentiated ALI epithelia [2,12].

Overall, this study contributes to the understanding of HCoV-OC43 replication in primary human bronchial epithelial ALI cultures and demonstrates that epithelial differentiation, enabled by culture of primary cells from patients at the ALI, is an important variable influencing HCoV-OC43 replication outcomes. In addition to confirming previous findings regarding replication kinetics, cell passage number, and virus inoculation times, we show that duration of ALI culture and infection route critically influence viral replication. In this study, we identified conditions for robust HCoV-OC43 replication in fully differentiated ALI cultures using a DoE-based screening approach. According to multivariable regression, maximal HCoV-OC43 quantities can be achieved when using passage 1 pHBECs, performing three sequential apical washes to remove mucus before apical infection or infecting via the basal route. We thereby contribute to the future standardization of virus infection protocols and provide a methodological basis for establishing infection models for other respiratory viruses that are difficult to propagate in conventional cell culture, such as human rhinovirus C and HCoV-HKU1.

## Figures and Tables

**Table 1:** Parameter settings maximizing HCoV-OC43 gRNA quantities according to the prediction profiler tool within the least-squares fit model within JMP, used for generating single-variable and interaction plots.

	Cell Lysate Samples	Apical Wash Samples
Passage number of pHBECs	1	1
Duration of ALI culture before infection [d]	21	21
Timepoint of last mucus wash relative to infection [d]	−4	0
Number of sequential mucus washes	3	3
Infection route	Basal	Basal
Duration of virus inoculation [h]	1	1
Interval between infection and harvest [d]	3	4

## Data Availability

All data reported in this paper will be shared by the lead contact upon request. Any additional information required to reanalyze the data reported in this paper is available from the lead contact upon request. Requests for further information and resources should be directed to the lead contact, Thomas Michler (thomas.michler@med.uni-muenchen.de).

## Supplementary Materials

The following supporting information can be downloaded at: https://www.mdpi.com/article/10.3390/cells15111010/s1, Figure S1: Data Processing and model fitting in JMP^®^ 18; Figure S2: Effect of ALI culture duration, infection route and virus dose on HCoV-OC43 replication in pHBECs from a second donor; Table S1: Effect of main variables and interactions on HCoV-OC43 gRNA levels: Cell lysate dataset; Table S2: Effect of main variables and interactions on HCoV-OC43 gRNA levels: Apical wash dataset; Table S3: JMP^®^ 18 design diagnostics; Table S4: Primer and probe sequences used for RT-qPCR analysis; Table S5: Cycling conditions used for RT-qPCR analysis; File S2: Experimental Design Matrix including Raw data; File S3: DoE Power analysis.
