# Supplementary material for: Multifactorial Analysis Identifies Conditions for Robust HCoV-OC43 Replication in Primary Human Bronchial Epithelial Cells Cultured at Air–Liquid Interface
Source: Cells. 2026 May 30;15(11):1010. doi: 10.3390/cells15111010 (PMC13256164; doi:10.3390/cells15111010)
Supplement: Supplementary file 1 [file cells-15-01010-s001.zip › Supplementary File_S1_Figures and Tables.pdf]

Supplementary data for manuscript:

Multifactorial Analysis Identifies Conditions for Robust HCoV-OC43 Replication in Primary Human Bronchial Epithelial Cells Cultured at Air–Liquid Interface

A

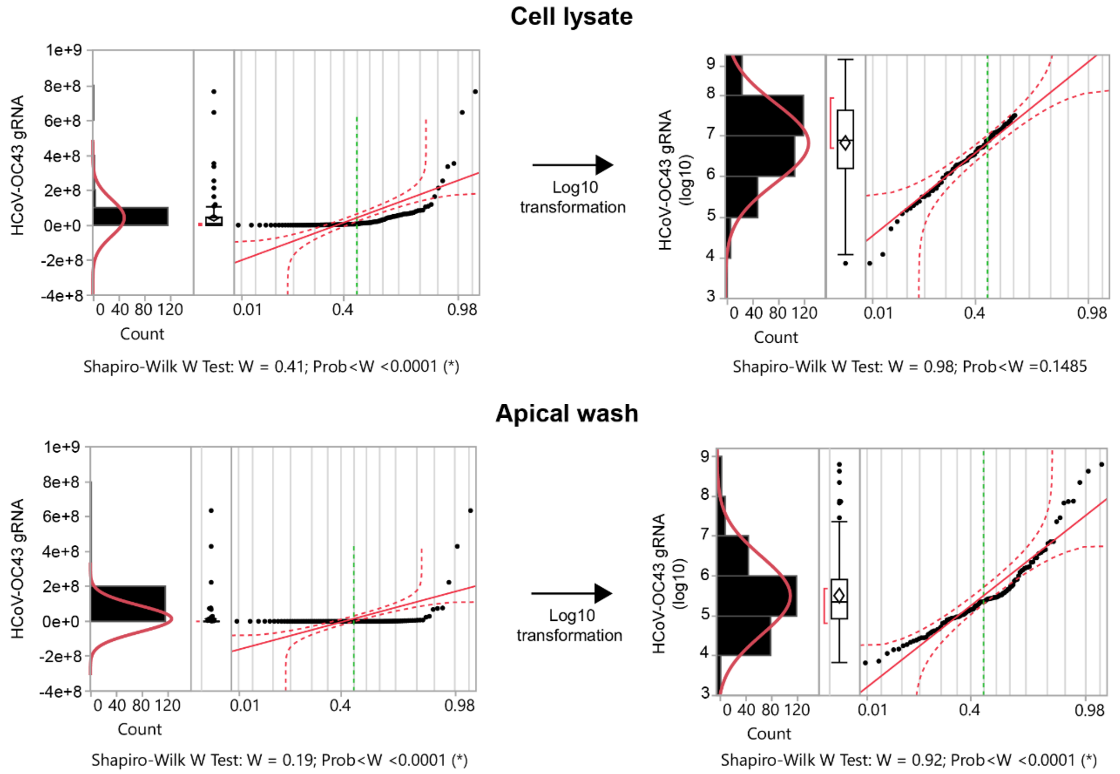

B

Least-squares fit regression model statistics

	Cell lysates		Apical washes	
	Before $\log_{10}$ transformation	After $\log_{10}$ transformation	Before $\log_{10}$ transformation	After $\log_{10}$ transformation
R^2	0.65	0.65	0.43	0.70
Adjusted R^2	0.52	0.51	0.21	0.59
RSME (root mean square error)	72039647	0.68	65191950	0.78
Lack of fit test	Prob > F < 0.0001 (*)	Prob > F = 0.37	Prob > F < 0.0001 (*)	Prob > F = 0.86
Number of observations included in model	120		119	

Supplementary Figure S1. Data Processing and model fitting in JMP® 18. (A) Distribution and normality of data points were assessed using histograms, normal quantile plots and the Shapiro-Wilk test. Analyses indicated non-normality for untransformed datasets (left panels). Log_{10} transformation significantly improved normality for cell lysate datasets, as indicated by non-significant Shapiro-Wilk test results, and improved normality for apical wash datasets (right panels). **(B)** Log_{10}-transformed data were subsequently analyzed using a least-squares regression model in JMP. Corresponding model fit statistics are shown.

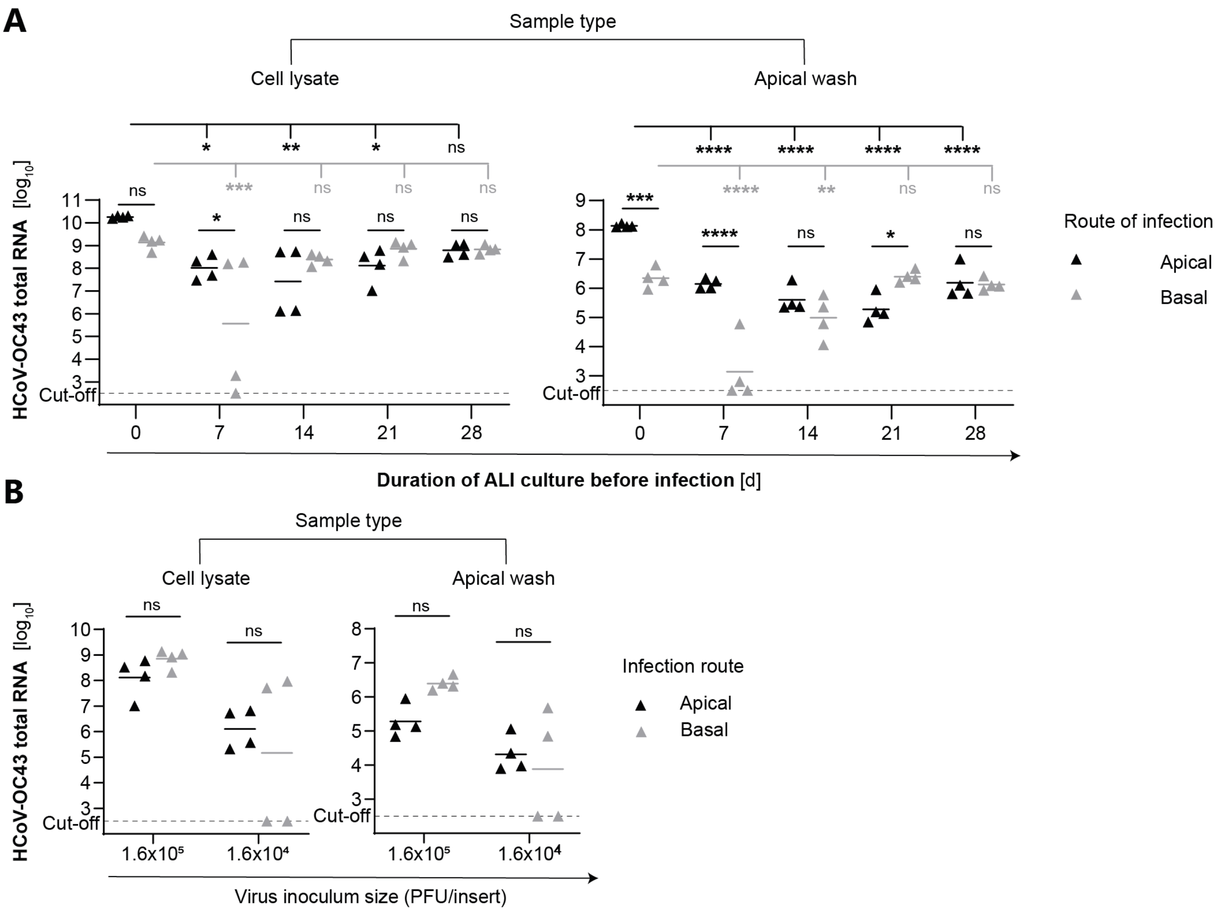

Supplementary Figure S2. Effect of ALI culture duration, infection route and virus dose on HCoV-OC43 replication in pHBECs from a second donor. pHBECs from another donor were cultured at ALI for 0-28 d and infected for 3 h via the apical or basal route. Viral RNA was harvested 3 d post-infection. Total viral RNA was quantified by RT-qPCR in cell lysate and apical wash sample compartments. **(A)** The effect of ALI culture duration and infection route on HCoV-OC43 total RNA quantities are shown. **(B)** pHBECs were cultured at the ALI for 21 d and were then infected with HCoV-OC43 at virus inoculum sizes of 1.6×10^5 or 1.6×10^4 PFU via the apical or basal route for 3 h. Viral RNA readouts were performed 3 d post-infection. **(A, B)** Values below the detection limit of RT-qPCR were set to cut-off ($\log_{10} = 2.5$), indicated by a dashed line. Data are presented as means ($n = 4$). Statistical analyses were performed using ordinary two-way ANOVAs followed by multiple comparisons tests. Šídák's test was used in (A) to assess differences between infection routes at each timepoint and in (B) to assess differences between infection route for each viral dose. Dunnett's test was used in (A) to compare each infection timepoint to d0. ns = not significant; *p < 0.05; **p < 0.01; ***p < 0.001; ****p < 0.0001.

Supplementary Table S1. Effect of main variables and interactions on HCoV-OC43 gRNA levels: Cell lysate dataset.

**Variable or variable interaction**	**Type of relationship modelled**	**p-value**
Duration of ALI culture before infection [d]	Linear	0.00025
Route of infection<br>+ Duration of ALI culture before infection [d]	Linear	0.00038
Interval between infection and harvest [d]	Quadratic	0.00352
Number of sequential mucus washes	Linear	0.03862

Supplementary Table S2. Effect of main variables and interactions on HCoV-OC43 gRNA levels: Apical wash dataset.

Variable or variable interaction	Type of relationship modelled	p-value
Duration of ALI culture before infection [d]	Quadratic	< 0.00001
Interval between infection and harvest [d]	Linear	0.00005
Route of infection	Linear	0.00009

Supplementary Table S3. JMP® 18 design diagnostics.

D Efficiency	39.6
G Efficiency	12.5
A Efficiency	23.2

Supplementary Table S4. Primer and probe sequences used for RT-qPCR analysis.

Primer/Probe Name	Target	Sequence (5'-3')
OC43-RdRP_fw	HCoV-OC43 RNA-dependent RNA polymerase (Nsp12)	TATGGTGGCTGGGATGATATGTTAC
OC43-RdRP_rev		AGGTTTGGCATAGCACGATCACACTT
OC43-RdRP_probe		FAM-ACAATCCTGTACTTATGGGTTGGGATTATCCT-BHQ1
OC43-NC_fw	HCoV-OC43 Nucleocapsid (NC)	CGA TGA GGC TAT TCC GAC TAG GT
OC43-NC_rev		CCTTCCTGAGCCTTCAATATAGTAACC
OC43-NC_probe		FAM- TCCGCCTGGCACGGTACTCCCT- BHQ1
hCCDC40_fw	Coiled-Coil Domain-Containing Protein 40 (necessary formotile cilia function) mRNA	CGGTCCACCAGAATCCAGAG
hCCDC40_rev		AGACGAACTCCTCCAGGTCT
hCCDC40_probe		FAM-TGGGCAGATTGACAGGATCCACAGA-TAMRA
hKRT5_fw	Keratin5 mRNA, which is part of the intermediate filaments of the cytoskeleton of basal epithelial cells	GCTGAGAGCCGAGATTGACA
hKRT5_rev		GGTCCAACTCCTTCTCCACTG
hKRT5_probe		FAM-TGTCAAGAAACAGTGCGCCAATCTGC-TAMRA
hMUC5AC_fw	Mucin glycoprotein MUC5AC mRNA, secreted by goblet cells	TGACGGGAAGCAATACACGG
hMUC5AC_rev		CAGCTCAGCCAGTACAGTGA
hMUC5AC_probe		FAM-TGCTGACCAAGCCCTGTGACAG-TAMRA
hSCGB1A1_fw	Secretoglobin Family 1A Member 1 (Uteroglobin) mRNA, secreted by club cells	GAAACTCGCTGTCACCCTCA
hSCGB1A1_rev		CTGCCTCCCTCATGTCTTG
hSCGB1A1_probe		FAM-TGCAGCTCCGCTTCTGCA-TAMRA
hGAPDH_fw	Glyceraldehyde-3-phosphate dehydrogenase mRNA, involved in metabolism	CCGTCAAGGCTGAGAACGG
hGAPDH_rev		CTCAGCGCCAGCATCGC
hGAPDH_probe		FAM-CATCTTCCAGGA/GCGAGATCCCTCC-TAMRA

Supplementary Table S5. Cycling conditions used for RT-qPCR analysis.

Cycle Step	Temp.	Time	Cycle No.
**PCR cycling conditions for HCoV-OC43 RdRP and NC**			
Reverse transcription (RT)	50°C	10 min	1
RT inactivation	95°C	3 min	1
Denaturation	95°C	15 s	45
Annealing/Extension	60°C	30 s	
**PCR cycling conditions for hCCDC40, hKRT5, MUC5AC, SCGB1A1, GAPDH**			
Reverse transcription (RT)	50°C	10 min	1
RT inactivation	95°C	3 min	1
Denaturation	95°C	15 s	45
Annealing/Extension	59°C	30 s	
